# Dynamic Covalent Boronic-Acid-Functionalized Alginate/PVA Hydrogels for pH and Shear-Responsive Drug Delivery

**DOI:** 10.3390/gels10080504

**Published:** 2024-07-31

**Authors:** Yessenia Oyarzún, José Ulloa, Matías Ceballos, Bruno F. Urbano

**Affiliations:** Departamento de Polímeros, Facultad de Ciencias Químicas, Universidad de Concepción, Concepción 4070386, Chile

**Keywords:** dynamic covalent bond, boronic acid, drug release

## Abstract

Herein, we investigated hydrogels composed of boronic-acid-functionalized alginate and blended with polyvinyl alcohol (PVA) of different molecular weights to control the release of metoclopramide hydrochloride as a function of pH and shear stress. The functionalization of alginate introduced dynamic covalent bonding and pH-responsive properties that can modulate network connectivity. The study investigated the viscoelastic properties of the hydrogels, their drug release profiles, and their responsiveness to changes in pH and shear forces. The results showed that a higher PVA molecular weight and alkaline pH conditions increased hydrogel viscosity and stiffness due to a more stable and interconnected network structure than acidic pH. Metoclopramide release revealed that the hydrogels exhibited pH-responsive drug release behavior. The drug was more readily released under acidic conditions due to the instability of sp^2^-hybridized boronate ester bonds. The influence of shear forces on the release of metoclopramide was also investigated at shear rates of 1, 10, and 100 s^−1^, revealing their effect on matrix stiffening. Research shows that AlgBA/PVA hydrogels have unique properties, such as dynamic covalent bonding, that make them sensitive to external mechanical forces. This sensitivity makes them ideal for applications where physiological conditions trigger drug release.

## 1. Introduction

Hydrogels have emerged as a standout biomaterial due to their unique properties, such as biocompatibility, biodegradability, flexibility, and ability to retain high amounts of water. Hydrogels have applications in various fields, including pharmaceuticals, agriculture, cosmetics, and the food industry [1,2,3]. A hydrogel is a 3D network of hydrophilic polymer chains that are crosslinked and insoluble. Hydrogels can respond to environmental stimuli such as temperature, pH, and ionic strength depending on the type of polymer used, the degree of crosslinking, and the specific interactions involved [4,5,6,7,8]. Thus, by modifying the applied stimuli, hydrogels’ physical and mechanical characteristics may be tailored [9,10]. Sodium alginate (Alg) has been the subject of extensive research due to its unique properties. Alginate is a natural polysaccharide derived from brown algae abundantly found on marine coasts worldwide [11,12]. Alginate has found valuable applications in pharmaceutical and biomedical fields due to its biocompatibility, biodegradability, and non-toxicity. The particular properties of this biopolymer are crucial in the fabrication of scaffolds for tissue engineering, dental printing, and manufacturing dressings for wounds, among other applications [13,14,15].

Alginate can be functionalized with boronic acid to allow the formation of dynamic covalent bonds (DCB) between the boronic acid and either 1,2 or 1,3-diols to form boronate esters [3,16,17]. Esters are pH-sensitive and can give origin to hydrogels with dynamic crosslinking capabilities; therefore, they are biomaterials with self-healing, stimulus responsiveness, and injectability properties. Dynamic covalent bonds have expanded the potential applications of alginate, particularly in controlled drug release [18,19].

The behavior of these bonds is regulated by a dynamic equilibrium dependent on pH and the pKa of the boronic acid. The pKa determines the structure–reactivity relationship that influences the application, formation, and stability of dynamic covalent bonds [20]. One of the most commonly used boronic acid compounds is phenylboronic acid (pKa 8.8), and its analogs, such as 3-acetamido phenylboronic acid (pKa 8.5), 2-formylphenyl boronic acid (pKa 7.5), and 3-pyridylboronic acid (pKa 4.0) [21,22]. The formation and stability of the DCB are favored when the pH of the medium is above the pKa of the acid. Under these conditions, the boronate ion with tetrahedral geometry (sp^3^ hybridization) forms a less strained boronate ester ring. Conversely, when the pH is below the acid’s pKa, the boronate ester becomes less stable and more reversible due to the higher strain of the cyclic ester when it adopts its flat trigonal form (sp^2^ hybridization) [16].

Several studies have investigated the functionalization of sodium alginate with boronic acids (AlgBA) and their potential uses, including bioadhesive and self-healing properties [17,23], injectability [24,25], and drug delivery capabilities [26,27].

Hydrogels are primarily used to develop controlled drug delivery systems, as well as enhancing and extending drug administration, while minimizing any associated side effects [28,29]. Hydrogels are proven to be an excellent material for controlled drug release due to their structural properties, such as porosity and degree of crosslinking, along with their ability to encapsulate substances and release them using various mechanisms [30,31]. Drug release from hydrogel networks may occur by different mechanisms, including diffusion and swelling. Another less-studied type of release mechanism is the application of mechanical stress [32,33,34]. This will be an exogenous mechanism if the stress is from mechanical forces, such as external magnetism or ultrasound, and an endogenous mechanism if the forces involved are compression, tension, or shear. Examples of the latter are compression forces on articular cartilage, tension forces on muscles, or shear forces on arteries [33]. Shear stress is one of the fundamental forces associated with blood flow. It plays an important role in the regulation of biomolecular processes, such as platelet aggregation and endothelial cell function [35]. Shear stress can also be encountered in other scenarios, such as injection, chewing, swallowing, and creams, and it varies in the shear rate applied.

The present study focuses on the release of metoclopramide hydrochloride (MTC) from hydrogels made of alginate functionalized with 4-aminophenyl boronic acid (AlgBA) and crosslinked with polyvinyl alcohol (PVA) of varying molecular weights. MTC is a drug used to treat nausea and vomiting associated with cancer therapy, pregnancy, migraine, and acute diabetic gastroparesis, regularly administered orally and by injection [36]. We hypothesize that the incorporation of boronic acid into alginate hydrogel crosslinked with PVA of different molecular weights can modulate drug release rates by altering the hydrogel’s viscoelastic properties in response to external pH levels and shear forces. This controlled delivery of metoclopramide hydrochloride (MTC) occurs due to the dynamic crosslinking created by the hydrogel network’s dynamic covalent bonding of boronate esters [37]. Our results show that this hydrogel provides a dynamic drug delivery system that can change its network connectivity in response to pH and evaluate the impact of shear forces on the release of metoclopramide.

## 2. Results and Discussion

### 2.1. Synthesis of Modified Alginate with 4-Aminophenyl Boronic Acid (AlgBA)

AlgBA was synthesized by activating the carboxylate groups of alginate using EDC, which facilitated the formation of amide bonds between carboxyl and amine groups. NHS was also used to enhance the stability of active intermediates in coupling reactions, forming active ester functional groups with carboxylates.

**Figure 1:**
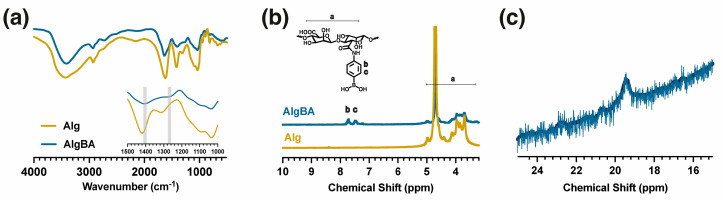
Spectroscopic characterization of boronic-acid-functionalized sodium alginate: (**a**) FTIR, (**b**) ^1^H−NMR (in D_2_O−d_2_) where “a” represents the protons of main chain and “b” and “c” are ascribed to the protons of the aromatic ring, and (**c**) ^11^B−NMR (in D_2_O−d_2_).

Figure 1 shows the spectroscopic characterization of AlgBA. The FT-IR spectra (Figure 1a) of sodium alginate and AlgBA show the characteristic bands of polysaccharides with carboxyl, carboxylate, and hydroxyl groups: a broad band at 3400 cm^−1^ (ν, O–H), 2925 cm^−1^ (ν, C–H), 1638 cm^−1^ (δ_as_, COO), 1409 cm^−1^ (δ_s_, COO), 1034 cm^−1^ (δ, O–C–O), and 1300 cm^−1^ (δ, C–C–H) The two spectra differ only in the presence of bands at 1450 and 1340 cm^−1^, associated with C–B and B–O bond stretching, respectively. Additionally, characteristic signals at 3.5 and 4.0 ppm correspond to the basic structure of the polymer in both spectra. At the same time, the peaks at 7.5 and 8.0 ppm are attributed to protons from phenyl groups (Figure 1b; AlgBA), which are absent in the alginate spectrum (Figure 1b; Alg). The ^11^B NMR spectra (Figure 1c) also displayed a peak at 19.37 ppm, indicating sp^3^ boron hybridization in AlgBA [38]. Inductively coupled plasma mass spectrometry (ICP-MS) was used to identify and quantify the boron content in AlgBA, which was found to be 0.86%, equal to a degree of substitution of 18%.

### 2.2. Preparation of AlgBA/PVA Hydrogels

**Figure 2:**
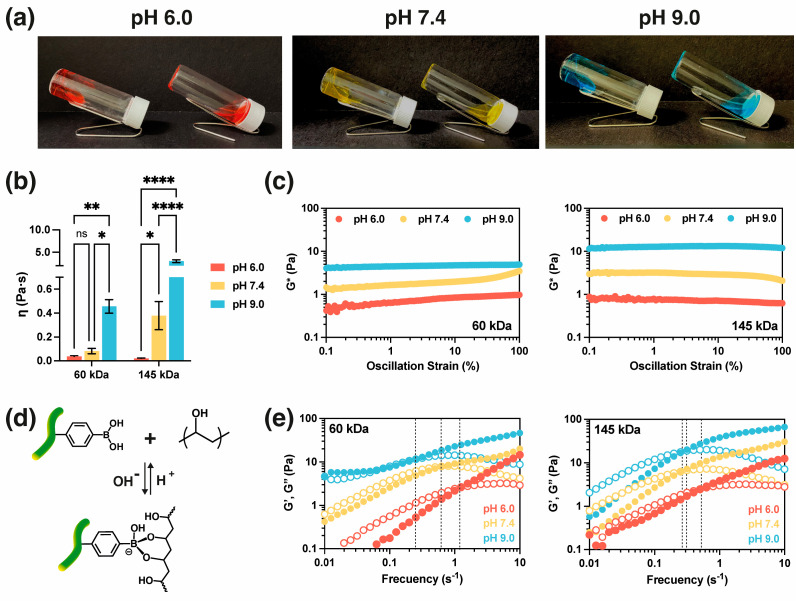
Rheological characterization of hydrogels. (**a**) Images of inversion test of hydrogel at different pH and PVA 60 kDa (right vial: AlgBA/PVA; left vial: control Alg/PVA); (**b**) viscosity of the hydrogels determined via flow test (*n* = 3) (statistical analysis: two−way ANOVA, Sidak test 95% confidence interval, * *p* < 0.0332; ** *p* < 0.0021; **** *p* < 0.0001; ns: non significant); (**c**) complex modulus (G*) of amplitude sweeps as function of pH (*n* = 3); (**d**) Scheme of formation of boronate ester between modified alginate (AlgBA) and poly(vinyl alcohol) (PVA); and (**e**) frequency sweeps of AlgBA/PVA displaying the crossover frequency related to relaxation time.

Hydrogels were formed by establishing dynamic covalent boronate ester bonds between AlgBA and PVA at different pH. A vial inversion test was used to confirm the formation of hydrogels (Figure 2a) qualitatively. The hydrogels were jellified immediately after homogenizing the mixture at the three pH levels studied. The solution containing boronic acid was observed to gel within the alginate structure (Figure 2a, left vial); conversely, the solution prepared with non-functionalized alginate flowed entirely after inversion of the vial (Figure 2a, right vial).

Rheometry was used to characterize the viscoelastic properties of the AlgBA/PVA hydrogels. The effects of pH and PVA molecular weight on the viscoelastic properties of the hydrogels were tested. Flow, amplitude, and frequency tests were measured, revealing that properties such as viscosity and the modulus are dependent on pH and PVA molecular weight. Figure 2b shows the obtained viscosity values, and a clear positive correlation between viscosity and pH was observed. Moreover, a positive correlation between PVA molecular weight and viscosity was also observed. For instance, AlgBA/PVA145 hydrogels displayed higher viscosity than AlgBA/PVA60 hydrogels at pH 7.4 and 9.0. The hydrogels were also characterized by an amplitude test (Figure 2c), which characterizes the rigidity of the hydrogel. In this type of analysis, the complex modulus, G*, describes the overall resistance of the material to deformation and includes the elastic (G′) and viscous (G″) contributions of a viscoelastic material according to the following equation $G^{*}=\sqrt{{\left(G^{\prime }\right)}^{2}+{\left(G^{\prime \prime }\right)}^{2}}$.

The influence of pH on the material’s stiffness was significant (Figure 2c). An increase in pH resulted in an evident rise in the complex modulus, indicating increased rigidity. The pH effect on the boronic acids is responsible for the changes in viscosity and complex modulus observed in the experiment. Boronic acid analogs, such as acrylamide phenyl boronic acid, have a pKa of 7.8 [39]. The most common structure at pH < pKa is sp^2^-hybridized boron with a planar trigonal boronate ester. On the other hand, at pH > pKa, the sp^3^ boron and tetrahedral boronate esters predominate (see Figure 2d). Our results showed that at pH 9.0, the hydrogels showed a significantly higher viscosity due to the formation of tetrahedral boronate ester bonds. This leads to a strongly interconnected network compared to hydrogels at pH 7.4 and 6.0. In contrast, at low pH, the stability of the boronate ester decreased, resulting in a hydrogel with increased fluidity and decreased viscosity [40]. The changes in the complex modulus, G*, are related to the stability of the dynamic covalent boronate ester bonds formed at different pH values [41]. As previously discussed, forming the more stable tetrahedral boronate ester is prevalent in alkaline conditions, creating highly interconnected networks. The stability, however, decreases as the pH decreases, primarily because the bond adopts a more prevalent trigonal conformation in neutral and acidic solutions [36].

Our data show that the increase in molecular weight of PVA leads to an increase in viscosity and complex modulus (Figure 2c). For example, at pH 9.0, the viscosity increases from 0.45 ± 0.05 Pa s to 3.02 ± 0.29 Pa s for PVA 60 kDa and 145 kDa, respectively. Similarly, the modulus varies by approximately 4.95 ± 3.04 Pa to 11.51 ± 5.20 Pa for PVA 60 kDa and 145 kDa, respectively. The increase in viscosity and the modulus with PVA molecular weight is due to increased crosslinking, which enhances chain interactions as the PVA molecular weight increases [40]. The greater availability of 1,3 *cis*-diols to form boronate esters and physical crosslinking due to chain entanglements and hydrogen bond formation can be attributed to the increase in network connectivity, considering that the concentration of AlgBA remains constant in both formulations.

Figure 2e shows the frequency dependence of the elastic (G′) and viscous (G″) modulus. At low frequencies, the viscous modulus predominates (G″ > G′), indicating a liquid-like behavior of the hydrogel. As the frequency increases, a crossover of the G′ and G″ lines (Figure 2e) indicates a transition towards a more solid-like behavior. The change in the modulus is attributed to the fact that at low frequencies, there is more time for the hydrogel chains to restructure in response to a disturbance, favoring chain movement and fluidity. Conversely, less time is available for polymer chain rearrangement at high frequencies, resulting in increased rigidity and a rise in elastic modulus [42]. The intersection frequency where the G′ and G″ curves meet (Figure 2c) is related to the relaxation time (τ_R_) of the hydrogel. The relaxation time (τ_R_) can be calculated using the equation τ_R_ = 2π/ƒ_c_ where ƒ_c_ is the crossover frequency. This value provides information about the stability of the dynamic covalent bonds (DCB) and the crosslinking network. Long relaxation times indicate a more stable network [41]. AlgBA/PVA60 displayed 5.0 s, 10.1 s, and 25.1 s relaxation times at pH 6.0, 7.4, and 9.0, respectively. On the other hand, AlgBA/PVA145 displayed a relaxation time of 12.6 s, 20.3 s, and 28.5 s at pH 6.0, 7.4, and 9.0, respectively (Figure 2e). The relaxation times increase as the pH increases. At pH 9.0, there are longer relaxation times than the ones observed at pH 6.0 and 7.4, indicating higher stability and the formation of more stable tetrahedral boronate esters. It is worth noting that the concentration of AlgBA is the same in both formulations; therefore, the concentration of boronic acid is the same. Increasing the molar mass of the PVA does not enhance the network’s connectivity through boronate esters. Instead, it only increases the sites of entanglements of the PVA chains. This explains why the increase in molar mass does not significantly affect the relaxation time. 

**Figure 3:**
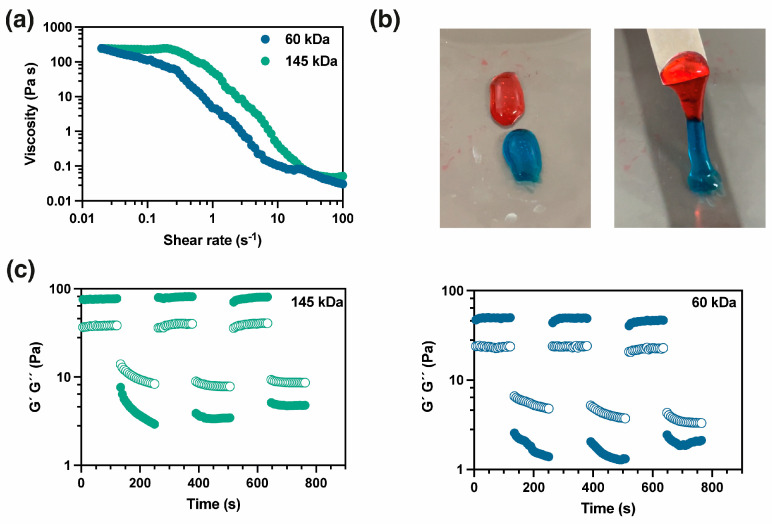
(**a**) Shear thinning test of the hydrogels; (**b**) images showing self−healing properties; (**c**) time sweep test cycles of 0.1% and 100% deformation.

The shear thinning properties of the hydrogel were investigated by flow testing. Figure 3a shows the viscosity profiles as a function of shear rate. Both hydrogels show similar zero shear viscosity (*ca.* 230 Pa·s); however, the hydrogel formulated with PVA 145 required a higher shear rate to reduce the viscosity, which is attributed to interactions between polymer chains due to the molar mass. To demonstrate the self-healing behavior of these hydrogels, Figure 3b qualitatively shows the self-repair properties of the hydrogel when two samples are put in contact. Additionally, time sweep tests were carried out to assess the recovery of the to the hydrogels. For this purpose, the hydrogels were subjected to three deformation cycles, applying 0.1% and 100% deformation. The results show that hydrogels possess a superior recovery capability, with both G′ and G″ remaining nearly constant across all cycles and demonstrating microstructure recovery.

**Figure 4:**
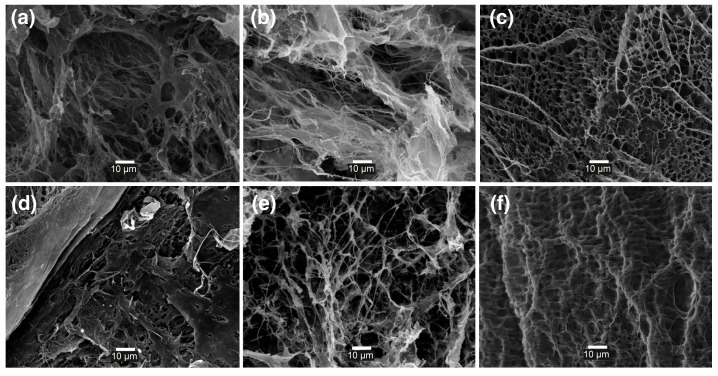
SEM images (scale 10 μm) of lyophilized hydrogels (**a**) AlgBA/PVA60 pH 6.0; (**b**) AlgBA/PVA145 pH 6.0; (**c**) AlgBA/PVA60 pH 7.4; (**d**) AlgBA/PVA145 pH 7.4; (**e**) AlgBA/PVA60 pH 9.0; (**f**) AlgBA/PVA145 pH 9.0.

Figure 4 shows the SEM images of the hydrogels at different pH values and molar masses of PVA. All the hydrogels showed an irregular porous polymer network structure; however, the hydrogels at pH 9.0 showed a more regular and less porous structure than their analogs obtained at more acidic pH, consistent with a more crosslinked structure.

### 2.3. Study of Metoclopramide Drug Release

**Figure 5:**
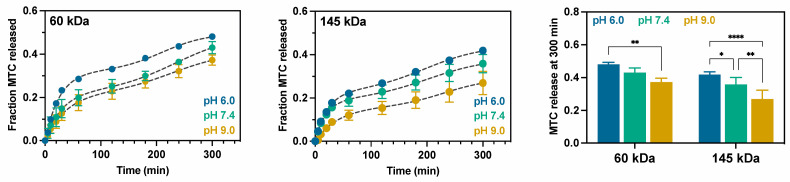
MTC release profile AlgBA/PVA60 and AlgBA/PVA145 as a function of pH. The segmented black lines of release curves correspond to the nonlinear fit of the Gallagher–Corrigan kinetic model. (statistical analysis: Two-way ANOVA, Sidak test 95% confidence interval; * *p* < 0.0332; ** *p* < 0.0021; **** *p* < 0.0001).

Release experiments were performed for AlgBA/PVA60 and AlgBA/PVA145 hydrogels at pH 6.0, 7.4, and 9.0. The selection of specific pH values for this study is intricately linked to the physiological interactions and stability of the complex in various media. A pH of 6.0 is chosen to simulate acidic environments commonly found in certain pathological conditions, such as inflamed or tumor tissues. The pH of 7.4 represents the typical physiological pH of blood and extracellular fluids under normal body conditions. Finally, the choice of pH 9.0 is related to the stability of the complex, as this pH is greater than the pKa of the boronic acid complex, ensuring the deprotonation of boronic acid and enhancing its binding affinity and stability in basic conditions. The results are presented in Figure 5, which shows that drug release occurred through diffusion within the hydrogel network, driven by a concentration gradient. The curves reveal changes in release behavior are observed with increasing pH. Alkaline pH decreases both the release rate and the amount of MTC released, while acidic pH favors the release of MTC. This behavior was observed for PVA with 60 and 145 kDa molecular weights.

The release profiles were analyzed using the Gallagher–Corrigan kinetic model (Equation (1)) [43]. This model consists of two terms: the first describes the release associated with fast solute diffusion to the solution, and the second term is attributed to the slower release occurring at longer times and associated with matrix degradation. Although the second phase is typically linked to carrier degradation, this model enables the quantification of both the rapid release stage and the loss of connectivity resulting from boronate ester breaks during the second stage. The equation describing the model is as follows:

(1) $$F=F_{\max}(1-e^{-k_{3}t})+(F_{\max}-F_{B})\left[\frac{e^{k_{4}t-k_{4}t_{\max}}}{1+e^{k_{4}t-k_{4}t_{\max}}}\right]$$

where *F* is the fraction released at time *t*, *F*_max_ indicates the maximum fraction of the drug released during the total time, *F_B_* is the fraction released in stage I, tmax indicates the time at which the maximal rate of the MTC release is accomplished during stage II, and *k*_3_ and *k*_4_ are the rate constants for stage I and stage II, respectively. Table 1 displays the kinetic parameters.

The model fitting reveals that increased pH decreases the maximum release (*F*_max_). Still, the release of MTC is not significantly different in the first stage (associated with the burst release). Furthermore, there are no significant differences in the rate constants (*k*_3_ and *k*_4_) at the pH and molar mass studied for PVA. The quantity and rate of drug release vary depending on the stability and degree of crosslinking in each hydrogel. An increase in pH results in a more strongly interconnected network due to the higher stability of the formed boronate ester, making drug release more difficult and reducing the amount of MTC released. On the other hand, the lower stability of the boronate ester at pH 6.0 and 7.4 results in a weakly interconnected network, which facilitates drug release.

Increasing the molecular weight of PVA increases the crosslinking density, which results in a more rigid and viscous hydrogel, as evidenced by the rheometry, consequently reducing the amount of MTC released.

**Figure 6:**
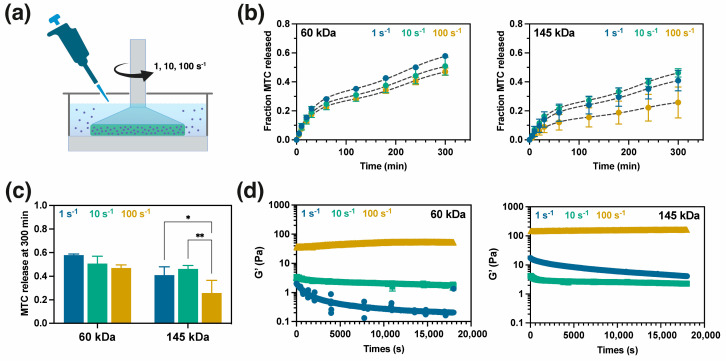
Release profile of MTC through shear forces. (**a**) Scheme of the system used to assess the MTC release in a rheometer, (**b**) cumulative MTC release from AlgBA/PVA60 and AlgBA/PVA145 hydrogels at pH 7.4, (**c**) MTC release at 300 min (statistical analysis: two−way ANOVA, Sidak test 95% confidence interval; * *p* < 0.0332; ** *p* < 0.0021), (**d**) change in storage modulus (G′) of hydrogels during the release studies.

To study the release of MTC under shear forces, AlgBA/PVA60 and AlgBA/PVA145 hydrogels were tested at pH 7.4 (Figure 6). Mechanical force is an important factor in evaluating drug release behavior in scenarios like injection, chewing, swallowing, and creams. In this sense, the experiment involved shear rates of 1, 10, and 100 s^−1^, which were associated with situations such as injections to chewing [44].

The AlgBA/PVA60 hydrogel and AlgBA/PVA145 hydrogel exhibited similar MTC release profiles (Figure 6b). However, the AlgBA/PVA145 hydrogel released less MTC than AlgBA/PVA60, especially at a low shear rate (Figure 6c). This difference is attributed to the higher molecular weight of PVA in the AlgBA/PVA145 hydrogel, which generates a network with increased connectivity obstructing the diffusion of MTC molecules. Interestingly, the amount of MTC released decreases as the shear rate increases, particularly for the 145 kDa PVA hydrogel (Figure 6c). To understand these results, we need to consider the changes experienced by the polymer network at different shear rates. An increase in frequency leads to an increase in storage modulus because it reduces the time available for the relaxation of the polymer chains, resulting in a stiffer hydrogel. Figure 6d shows the evolution of G′ during the release experiment. At a low frequency of 1 s^−1^, it is observed that there is a decrease in G′ as the experiment progresses. This decrease can be attributed to the relaxation of the AlgBA and PVA chains, which may explain the higher release observed at a shear rate of 1 s^−1^. At shear rates of 10 and 100 s^−1^, G′ increases in magnitude and remains constant throughout the experiment. This suggests that the microstructure of the hydrogel is maintained during the experiment. The increase in modulus at higher shear rates maintains a more intricate network connectivity, hindering the diffusion of the metoclopramide.

## 3. Conclusions

The present study aimed to investigate the use of hydrogels composed of boronic-acid-functionalized alginate (AlgBA) and blended with polyvinyl alcohol (PVA) of varying molecular weights to regulate the release of metoclopramide hydrochloride (MTC). The functionalization of alginate introduced dynamic covalent bonding and pH-responsive properties that can be leveraged to modulate network connectivity. The viscoelastic properties of hydrogels, their drug release profiles, and their responsiveness to changes in pH and shear forces were analyzed. The investigation revealed that the increased molecular weight of PVA and alkaline pH conditions led to increased hydrogel viscosity and rigidity, attributed to a more stable and interconnected network structure than acidic pH. The study also demonstrated that the hydrogels exhibited pH-responsive drug release behavior, where the drug was released more easily under acidic conditions due to the instability of sp2-hybridized boronate ester bonds.

Furthermore, the study investigated the impact of shear forces on the release of metoclopramide at shear rates of 1, 10, and 100 s^−1^, revealing their influence on the rigidization of the matrix. The research highlights that AlgBA/PVA hydrogels possess unique properties, such as dynamic covalent bonding, that make them sensitive to external mechanical forces. This sensitivity makes them advantageous for applications where physiological conditions trigger drug release.

## 4. Materials and Methods

### 4.1. Reagents

The materials used in the study include commercial sodium alginate (Alg, M/G ratio of 1.56, 15–25 cP at 1% in H_2_O, Sigma-Aldrich, Darmstadt, Germany), polyvinyl alcohol with molecular weights of 60 and 145 kDa (PVA, fully hydrolyzed, Merck), 2-(N-morpholino) ethanesulfonic acid buffer solution (MES, 99% titration, Sigma-Aldrich), phosphate-buffered solution at pH 6.0 (Na_2_HPO_4_/NaH_2_PO_4_, 0.01 mol L^−1^), phosphate-buffered saline solution at pH 7.4 (PBS, Sigma-Aldrich, 0.01 mol L^−1^), carbonate buffer solution at pH 9.0 (Na_2_CO_3_/NaHCO_3_, 0.01 mol L^−1^), sodium hydroxide (NaOH, 99%, Merck), 1-(3-dimethylaminopropyl)-3-ethylcarbodiimide hydrochloride (EDC·HCl, 98%, Sigma-Aldrich), N-hydroxysuccinimide (NHS, 98%, Sigma-Aldrich), 4-aminophenylboronic acid hydrochloride (95%, AK Scientific), triethylamine (TEA, Sigma-Aldrich), and metoclopramide hydrochloride (MTC·HCl, Sigma).

### 4.2. Synthesis of Modified Alginate with 4-Aminophenyl Boronic Acid (AlgBA)

Firstly, 1.0 g of sodium alginate was dissolved in 100 mL of MES buffer (0.1 mol L^−1^) with continuous stirring at room temperature. The pH was adjusted to 5.5 by adding NaOH (2 mol L^−1^). Then, EDC (700 mg, 4 mmol), NHS (300 mg, 0.86 mmol), and 4-aminophenylboronic acid (400 mg, 3 mmol) were added. The mixture was stirred constantly at room temperature for 24 h. Purification was conducted through 7 days of dialysis (membranes of 14 kDa), and the solution was finally lyophilized (Telstar Lyoquest-55, Barcelona, Spain) to obtain the dried product.

### 4.3. Preparation of AlgBA/PVA Hydrogels

The hydrogels were obtained by mixing solutions of AlgBA and PVA in a 3:1 ratio (AlgBA:PVA) at pH 6.0, 7.4, and 9.0 (pH ± 0.1). For this purpose, AlgBA and PVA solutions with variable molar mass weights (60 kDa and 145 kDa) were prepared at a concentration of 1.5% *w*/*v*, dissolving the polymer in a PBS buffer adjusted to pH 6.0, 7.4, and 9.0 (0.01 mol L^−1^). PVA solutions were stirred at 80 °C for 3 h until completely dissolved. Subsequently, the AlgBA solution was mixed with the PVA solution and vortexed for 30 s to form and homogenize the hydrogel. The formation of the hydrogel was evaluated through an inversion test, comparing gelation with a control hydrogel obtained by mixing a solution of commercial sodium alginate with PVA (Alg:PVA) in the same proportions and concentrations mentioned earlier.

The encapsulation of MTC was carried out by mixing solutions of AlgBA, MTC, and PVA in Eppendorf tubes in ratios of 3:0.08:1 (AlgBA:MTC:PVA), obtaining a total volume of 1 mL of hydrogel. This encapsulation was performed in situ by adding a 1% *w*/*v* concentration of the drug dissolved in PBS buffer (pH 6.0, 7.4, and 9.0) to the AlgBA solution before the hydrogel formation. Subsequently, PVA was added to achieve a final volume of 1 mL, and the mixture was stirred for complete homogenization.

### 4.4. MTC Release Studies

The release assays were conducted using conventional agitation and shear-induced stress. Precisely, MTC release from AlgBA/PVA-MTC hydrogels at pH 6.0, 7.4, and 9.0, as well as PVA of 60 kDa and 145 kDa, was measured. To perform the assay, 1 mL of AlgBA/PVA-MTC was mixed with 500 µL of buffer and placed in an orbital shaker (Heidolph, model Unimax 1010) at 37 °C, stirring constantly at 50 rpm and in triplicate. The study extracted 300 µL aliquots of the supernatant at different time intervals after centrifugation (Witeg, CF-10) for 5 to 300 min. The same volume was replaced with a fresh buffer to maintain a constant volume.

The drug release from AlgBA/PVA-MTC hydrogels at pH 7.4 and PVA with molecular weights of 60 kDa and 145 kDa was conducted at 37 °C using a parallel plate rheometer equipped with a submersion device and covered to avoid the solvent evaporation. A 40 mm plate was used with a 200 µm gap, shear rates of 1, 10, and 100 s^−1^, 1% strain, and an oscillation time of 5 h. For this procedure, 260 µL of a drug-loaded hydrogel (AlgBA/PVA-MTC) was placed between the upper plate and the Peltier plate, which were then immersed in 25 mL of PBS buffer solution at pH 7.4. Samples of 1 mL were taken at different time intervals (ranging from 5 to 300 min), and the same volume was replaced with fresh buffer to maintain a constant volume. A solvent trap prevented solution evaporation during the drug release monitoring process.

UV–Vis spectrophotometry (ThermoScientific, Waltham, MA, USA, Orion AquaMate 8000) was used to monitor the drug release at a wavelength of 309 nm. Calibration curves for MTC at pH 6.0, 7.4, and 9.0 were constructed using solutions with MTC concentrations ranging from 3.08 µg mL^−1^ to 37 µg mL^−1^.

### 4.5. Characterization Techniques

Fourier-transform infrared spectroscopy (FTIR) characterized the lyophilized product (AlgBA) on a NICOLET Magna 550 spectrophotometer. Samples were prepared by forming a solid solution with KBr and analyzed in the mid-infrared range of 400–4000 cm^−1^. A sample of commercial sodium alginate was also characterized as a control sample.

Nuclear magnetic resonance (NMR) was used to characterize AlgBA further using ^1^H and ^11^B NMR. A solution of the lyophilized product and sodium alginate in deuterated water (D_2_O) at a concentration of 10 mg mL^−1^ was prepared; subsequently, the samples were analyzed using a Bruker Avance III HD 400 MHz instrument.

Inductively coupled plasma (ICP) was used to analyze the boron content. The ICP-MS ThermoScientific model iCAP RQ was used to analyze an aqueous solution containing 15 mg of AlgBA. 

Rheometric analysis was also performed. The viscoelastic properties of AlgBA/PVA hydrogels were evaluated at pH 6.0, 7.4, and 9.0 using rheometry on a TA Instruments DHR-3 parallel plate rheometer. The hydrogels were loaded onto the rheometer, equipped with a Peltier plate for temperature control. Oscillatory strain and frequency tests were conducted at 37 °C using a rough-surfaced upper plate with a diameter of 20 mm and a gap of 200 µm, in triplicate, and with a solvent trap to prevent water evaporation. The oscillatory strain sweep tests were conducted within a range of 0.01% to 500% strain at a frequency of 1 s^−1^ to determine the storage modulus (G′), loss modulus (G″), and the linear viscoelastic region of the hydrogels. Viscosity tests were conducted at a 1% strain, determined from the linear viscoelastic region, with a shear rate of 50 s^−1^ for 120 s. Frequency sweep tests were conducted from 0.01 to 100 s^−1^ at a 1% strain to evaluate the hydrogels’ behavior. A flow test was performed at pH 7.4 and 37 °C for 5 h, with a strain rate of 1%, at frequencies of 1, 10, and 100 s^−1^. 

Scanning electron microscopy (SEM): The surface morphology of AlgBA/PVA hydrogels at different pH values and PVA molecular weights was studied using SEM. The hydrogels were frozen, lyophilized, and then analyzed using a VEGA3 Easyprobe SBU (TESCAN) scanning electron microscope to capture images and evaluate the porosity features.

## Figures and Tables

**Table 1:** The MTC kinetic release parameters obtained from the Gallager–Corrigan model.

		60 kDa			145 kDa	
	pH 6.0	pH 7.4	pH 9.0	pH 6.0	pH 7.4	pH 9.0
*F*_max_	0.318 ± 0.007	0.242 ± 0.026	0.226 ± 0.022	0.226 ± 0.010	0.190 ± 0.034	0.156 ± 0.401
*F_B_*	0.140 ± 0.024	0.030 ± 0.024	0.040 ± 0.031	0.009 ± 0.027	0.016 ± 0.016	0.018 ± 0.034
*k*_3_	0.038 ± 0.002	0.032 ± 0.015	0.025 ± 0.007	0.044 ± 0.003	0.051 ± 0.014	0.025 ± 0.004
*k*_4_	0.025 ± 0.003	0.021 ± 0.010	0.020 ± 0.001	0.019 ± 0.002	0.018 ± 0.001	0.022 ± 0.004
*t*_max_	208.625 ± 7.364	231.450 ± 8.655	233.233 ± 18.551	196.850 ± 7.445	201.200 ± 6.969	233.800 ± 5.631

## Data Availability

All data and materials are available upon request from the corresponding author. The data are not publicly available due to ongoing research using a part of the data.
